# Detecting accelerometer non-wear periods using change in acceleration combined with rate-of-change in temperature

**DOI:** 10.1186/s12874-022-01633-6

**Published:** 2022-05-20

**Authors:** Adam Vert, Kyle S. Weber, Vanessa Thai, Erin Turner, Kit B. Beyer, Benjamin F Cornish, F. Elizabeth Godkin, Christopher Wong, William E. McIlroy, Karen Van Ooteghem

**Affiliations:** Department of Kinesiology and Health Sciences, Faculty of Health, University of Waterloo, 200 University Ave, Waterloo, ON N2L 3G1 Canada

**Keywords:** Accelerometry, Physical activity, Remote monitoring, Wearables, Non-wear detection, Behavior classification, Algorithm

## Abstract

**Background:**

Accelerometery is commonly used to estimate physical activity, sleep, and sedentary behavior. In free-living conditions, periods of device removal (non-wear) can lead to misclassification of behavior with consequences for research outcomes and clinical decision making. Common methods for non-wear detection are limited by data transformations (e.g., activity counts) or algorithm parameters such as minimum durations or absolute temperature thresholds that risk over- or under-estimating non-wear time. This study aimed to advance non-wear detection methods by integrating a ‘rate-of-change’ criterion for temperature into a combined temperature-acceleration algorithm.

**Methods:**

Data were from 39 participants with neurodegenerative disease (36% female; age: 45–83 years) who wore a tri-axial accelerometer (GENEActiv) on their wrist 24-h per day for 7-days as part of a multi-sensor protocol. The reference dataset was derived from visual inspection conducted by two expert analysts. Linear regression was used to establish temperature rate-of-change as a criterion for non-wear detection. A classification and regression tree (CART) decision tree classifier determined optimal parameters separately for non-wear start and end detection. Classifiers were trained using data from 15 participants (38.5%). Outputs from the CART analysis were supplemented based on edge cases and published parameters.

**Results:**

The dataset included 186 non-wear periods (85.5% < 60 min). Temperature rate-of-change over the first five minutes of non-wear was − 0.40 ± 0.17 °C/minute and 0.36 ± 0.21 °C/minute for the first five minutes following device donning. Performance of the DETACH (DEvice Temperature and Accelerometer CHange) algorithm was improved compared to existing algorithms with recall of 0.942 (95% CI 0.883 to 1.0), precision of 0.942 (95% CI 0.844 to 1.0), F1-Score of 0.942 (95% CI 0.880 to 1.0) and accuracy of 0.996 (0.994–1.000).

**Conclusion:**

The DETACH algorithm accurately detected non-wear intervals as short as five minutes; improving non-wear classification relative to current interval-based methods. Using temperature rate-of-change combined with acceleration results in a robust algorithm appropriate for use across different temperature ranges and settings. The ability to detect short non-wear periods is particularly relevant to free-living scenarios where brief but frequent removals occur, and for clinical application where misclassification of behavior may have important implications for healthcare decision-making.

**Supplementary Information:**

The online version contains supplementary material available at 10.1186/s12874-022-01633-6.

## Background

There has been tremendous growth in the use of wearable sensor technologies as a method to evaluate fitness and health [1–4] and in particular, the use of accelerometry to assess physical activity volume and intensity [5–12]. In addition to evaluating activity, there is critical need to assess other daily behaviors such as sleep [6, 13], and sedentary time [5, 6, 9, 12, 13] which are characterized by a relative absence of movement. Quantifying and characterizing these activities is emerging as critical for understanding disease risk and improving health outcomes [6, 14, 15]. The unique advantage of accelerometry is its ability to capture data continuously in unsupervised, free-living conditions [16]. During free-living wear however, there are circumstances that can pose challenges to data analysis such as periods of device removal (non-wear) and the potential problem of misclassifying these periods as sleep or sedentary behavior due to the shared feature of an absence in movement. Appropriate classification of non-wear not only ensures that behaviors are properly represented but also allows for the use of non-wear time as a measure of compliance to device wear and thus, device useability. For example, non-wear can be used to evaluate factors that impact compliance such as removal due to interference with daily activities (e.g., removing a device to avoid contact with water) [7, 10], discomfort [10], or forgetfulness with respect to donning the device after a removal [10, 17]. In short, failure to accurately identify non-wear periods affects downstream measures, including the volume of valid, usable data, and amount of sleep, sedentary behavior and activity estimates [5, 7, 17–19]. As such, there is a need for validated, low-burden, automated methods to identify periods of non-wear time [20] for accelerometer-based devices [21].

Despite the importance of accurate non-wear detection for remote health monitoring, current device capabilities have noticeable limitations. Wearable devices that measure biopotentials, skin conductance and/or light refraction (e.g., oximetry) can rely on signal discontinuity due to a lack of skin contact to detect non-wear periods. However, most low-cost, wearable accelerometers and gyroscopes used to assess health behaviors in clinical and research settings must rely on a lack of change in the signal to indicate a period-of non-wear and in devices where there is only one signal-type available (e.g., acceleration only), non-wear can be difficult to resolve. For example, ‘activity counts’ are commonly used to classify activity intensity from accelerometry [13], but the method of deriving non-wear time from the sum of consecutive ‘zero’ activity counts (time intervals during which there is little to no acceleration) can lead to misclassifying sleep or sedentary time as non-wear [9, 17, 18, 20, 22–24]. Further, some activity count algorithms are device-specific which limits their utility, and any motion artifacts present during non-wear (e.g., external vibrations registered by the device when it is resting on a surface) can result in mistakenly identifying them as activity [23, 25]. For these reasons, an increasing number of algorithms are being developed to determine non-wear time from raw acceleration data (e.g., [11], [25]).

Non-wear algorithms applied to raw acceleration data typically consider pre-defined, minimum durations required for a period to be considered non-wear, and specific parameters for determining the start and end of a non-wear period [7, 11, 26]. This pre-defined duration, called window length, is a key factor affecting downstream measures of total wear time [9, 18, 24], number of non-wear periods [9], sedentary time [9, 24], and estimates of physical activity levels [22, 27]. The most common window length reported in the literature is 60 minutes [11, 12, 19]. While 60 minutes ensures that large periods of non-wear are not misclassified as wear time, work has shown that most non-wear periods are shorter than 60 minutes (e.g., removal for a shower) and therefore, it is likely that a significant number of non-wear periods are missed using this 60-minute window length [16, 26]. The risk associated with using non-wear algorithms that require a longer window length is an overestimation of wear time and sedentary time [9, 12]. Alternatively, using a shorter window length increases the potential for sleep and sedentary behavior to be misclassified as non-wear time [24]. To safeguard against these possibilities, data from additional sensors embedded in the device (e.g., light, temperature) can be used to supplement accelerometry data for non-wear detection. For example, devices with temperature sensors can use a threshold for non-wear that accounts for the decrease in temperature that occurs when the accelerometer is not in contact with the body. Zhou et al. [25] developed a non-wear algorithm with an absolute minimum temperature threshold of 26 °C as a criterion for non-wear combined with a threshold for standard deviation of acceleration. The additional temperature criterion improved sensitivity and specificity compared to an acceleration-only algorithm [25]. However, the use of an absolute threshold for temperature leads to the possibility that the start of the non-wear period is missed due to the time required for temperature to decrease once the device has been removed. More recently, Syed et al. [28] used a deep learning approach to detect device removal and subsequent donning to identify the start and end of non-wear periods from accelerometry, and similar to [25], the method does not rely on a threshold for window length. Although the study demonstrated improved performance compared to existing window-based algorithms, deep learning methods may be limited by the need for retraining to meet different use cases, and challenges to continued development and implementation among users less experienced with machine learning methods.

Recently our group evaluated adherence to daily device-wearing for remote health monitoring in older adults and persons living with neurodegenerative disease (NDD) by examining volume and patterns of non-wear [16]. Specific interest in the clinical application of wearables to aging and NDD, is driven by the need to understand daily health-related behaviors in the context of advancing age or disease to aid in health-care decision making. Accurate non-wear detection is critical for ensuring clinical utility of remote health assessment since misclassification of behaviors can have significant consequences for activity prescription and monitoring for treatment adherence and effectiveness. For example, recent evidence suggests that the *pattern* of sedentary behavior, in addition to volume, is linked to physical function, cardiorespiratory fitness [29], and metabolic outcomes [30, 31], emphasizing the risk of misclassifying non-wear periods as sedentary behavior. In older adults and persons with NDD, increased sedentary time or motor symptoms such as akinesia or rigidity in Parkinson’s disease, or hemiplegia following stroke, can complicate accelerometry-derived behavior classification (non-wear, sedentary behavior, or manifestation of disease-specific symptoms). These concerns are specifically important because small changes in behavior can be clinically meaningful with respect to disease tracking and evaluation of treatment effectiveness [32, 33].

The current work aimed to advance existing interval-based methods by building on previously published non-wear algorithms [11, 25]. Specifically, the study implemented a combined temperature and acceleration (CTA) algorithm with a *rate-of-change* criterion for temperature to identify the start and end of non-wear periods through the detection of device temperature and acceleration changes (DETACH) characteristic of these events. The first objective was to optimize parameters for non-wear start and stop time detection, window length and the temperature criterion, to improve the accuracy of non-wear classification. The second objective was to compare the algorithm performance against the current standards for non-wear detection including algorithms developed by van Hees et al. [11] and the CTA developed by Zhou et al. [25]. All algorithms were compared to a manually labelled non-wear reference dataset from a cohort of persons diagnosed with a range of NDDs. It was hypothesized that the DETACH algorithm would perform better at detecting non-wear periods with shorter intervals than those reported to date, as reflected by standard performance measures of accuracy, precision, recall, and harmonic mean (F1). The DETACH algorithm has the potential to improve activity estimates and enable more accurate measures of compliance during free-living wear periods.

## Methods

### Study approach

This study used a combination of regression analysis and machine learning for DETACH algorithm development inclusive of temperature rate-of-change, followed by a comparison of the algorithm to published non-wear detection methods to evaluate performance. Specifically, phase one of the study used linear regression to establish the viability of, and criteria for, temperature rate-of-change as a parameter for identifying device removal. Phase two used a decision tree classifier to determine the optimal series of features, and their respective thresholds, for determining the start and end of a non-wear period. Following this phase, edge cases identified from the training data were used to establish additional algorithm rules. Finally, outputs from the finalized DETACH algorithm and from the van Hees [11] and Zhou [25] algorithms were compared using accuracy, precision, recall, and F1 score performance metrics.

### Data source

Data were collected as part of the Remote Monitoring in Neurodegenerative Disease (ReMiNDD) study conducted by the Ontario Neurodegenerative Disease Research Initiative (ONDRI) [34] which included 39 participants with a confirmed diagnosis of cerebrovascular disease, Alzheimer’s disease/amnestic mild cognitive impairment, frontotemporal dementia, Parkinson’s disease, or amyotrophic lateral sclerosis (36% female; age range: 45–83 years). Detailed descriptions of study participants and protocol are provided in [16].

### Data collection

Briefly, the study consisted of a baseline clinic visit to Sunnybrook Hospital in Toronto, Canada, a 7-day remote monitoring period using wearable technology, and an in-person discharge visit. Data collection took place from May 2019 to March 2020. Participants were instrumented with five wearable devices located bilaterally on the wrists and ankles, and on the chest. Participants were asked to wear the devices for 24-h per day except during bathing and swimming. Limb-worn devices were GENEActiv Original [35] which contain tri-axial accelerometers, a near-body temperature sensor, and a light sensor. The limb devices were mounted on the wrists using rubber watch straps and on the ankles with custom-made, medical-grade wraps [36]. Accelerometer data were collected at a sampling rate of 75 Hz with a dynamic range of ±8 g (1 g = 9.81 m/s^2^) and temperature data at a sampling rate of 0.25 Hz. The temperature sensor was accurate to +/− 1 degree Celsius [37].

### GENEActiv data processing

Data from one wrist device was used for each participant since the wrist is a common wear location for activity and sleep studies e.g. [38, 39] and was used in the comparator studies [11, 25]. Specifically, the left wrist data were used which represented the non-dominant limb for 95% of participants. Data consisted of six continuous 24-hour periods extracted from the 7-day protocol. For two participants, data volume was less than the six 24-h periods due to early discharge from the study (142/144 hours; 113/144 hours).

The raw GENEActiv accelerometer data were stored in standard gravity units for subsequent analysis. These data files were converted from compressed binary files into standardized European Data Format (EDF) using Python package PyEDFlib [40] and stored by sensor type (tri-axial accelerometer, temperature, light) as part of a standard data management process. Data files were also cropped to the end of data collection (i.e., final device removal) as determined by study logs combined with visual inspection of the data. Due to the low sampling frequency of the temperature signal, temperature data were smoothed using a 2nd order, low pass, Butterworth filter with a cut-off of 0.005 Hz prior to subsequent analysis, except when re-creating the algorithm developed by [25], where a moving average model was used to smooth the temperature data. No conditioning of accelerometer data took place, consistent with the work of both [11] and [25] . For the rate-of-change in temperature parameter used in the DETACH algorithm, a one-minute rate-of-change was used by calculating the difference of smoothed temperature values one minute apart. To submit an equivalent number of datapoints for accelerometer and temperature data to the decision tree classifiers, a one-minute rolling standard deviation of the accelerometer data was determined, and this output was down sampled from 75 Hz to 0.25 Hz.

### Reference dataset

**Fig. 1:**
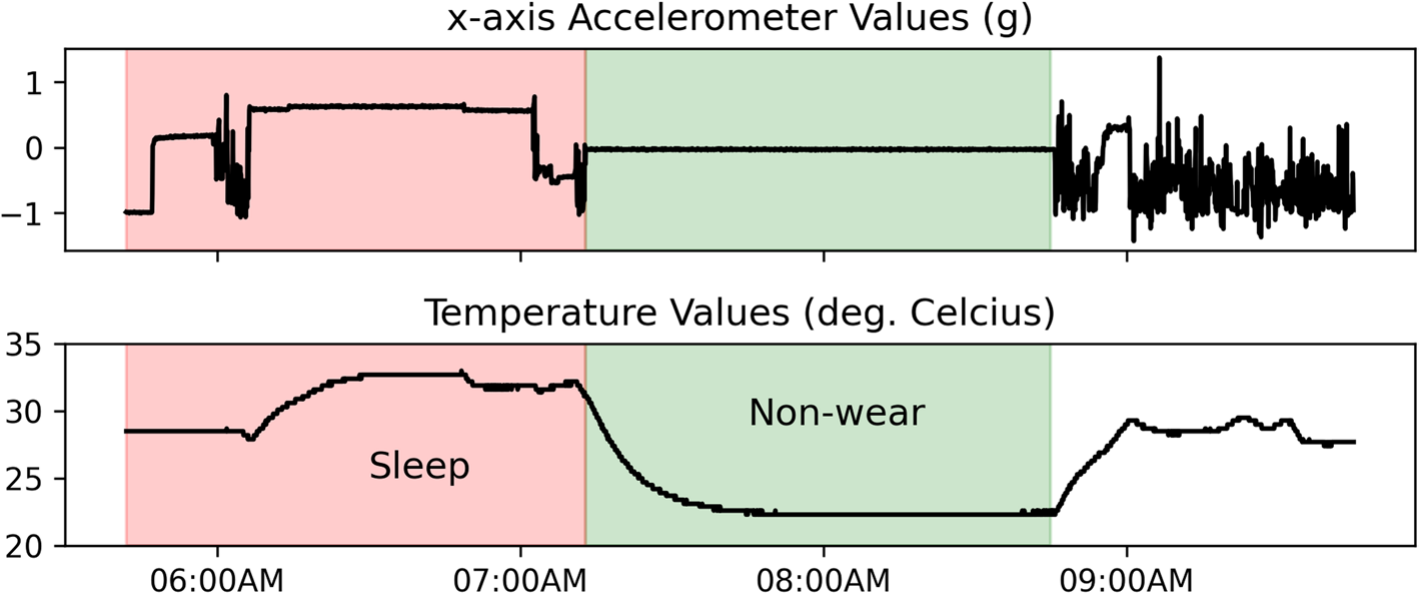
Sample temperature profile for sleep versus non-wear

The reference dataset used to evaluate the DETACH algorithm was based on visual inspection of non-wear periods conducted independently by two expert analysts using raw temperature and accelerometer data, with each assigned to reviewing data from half of the participants. Non-wear detection criteria included the absence of acceleration and a sustained, decreasing temperature (non-wear start) and the presence of acceleration with an accompanying increase in temperature (non-wear end). These changes in temperature, both in rate and direction, were distinguished from temperature changes that can be associated with sleep (Fig. 1). Prior to inspecting and annotating the reference dataset, analysts were trained on a known dataset by independently identifying probable non-wear periods, then reviewing and resolving discrepancies with the assistance of the study team. The known dataset was obtained from 2-day collections using GENEActiv accelerometers worn on the wrist. Each participant completed a series of structured (within any two-hour window) and unstructured (time of their choosing) removals, varying in length from one to 15 minutes, with instruction to remove as needed outside of these procedures. Non-wear time accounted for an average of 6 ± 14% of the collection period with a median duration of 5 minutes (range: 1 to 596 minutes). Following training, analysts independently annotated the reference dataset with any uncertainties resolved via consensus using device removal logs as reference. Removal logs were completed by study participants or their enrolled caregiver study partner who were asked to record what sensors were removed, the time of removal and re-attachment, and the reason for removal, without delay. Non-wear start and stop times were recorded with one-minute precision.

### Comparison algorithms

For both the van Hees [11, 41] and Zhou [25] algorithms, each data point from the raw acceleration signal is classified as wear or non-wear. Only the Zhou algorithm [25] uses temperature in addition to acceleration. The van Hees algorithm [41] only examines acceleration in 60-minute overlapping windows (15-minute steps, 45-minute overlap). Each window is classified as non-wear if the standard deviation of acceleration is less than 13 mg (1 mg = 0.00981 m/s^2^) or the acceleration range of that window is less than 50 mg in at least two of the three accelerometer axes. To remove implausible wear periods, a secondary condition is applied which classifies a detected wear period shorter than six or three hours as non-wear if it is less than 30% or 80% (respectively) of the combined duration of bordering non-wear periods [11].

The Zhou algorithm [25] examines both temperature and acceleration over a one-minute moving window. Each window is classified as non-wear if the mean temperature is less than or equal to 26 °C and the standard deviation of acceleration for each of the three axes is less than 13 mg. A secondary condition is also used to identify non-wear when the temperature is below 26 °C but the accelerometer standard deviation criterion is not met. In this instance, if the temperature in the current window is lower than the mean temperature of the previous one-minute window, the current window is labeled non-wear.

### Establishing temperature rate-of-change as a non-wear feature

**Fig. 2:**
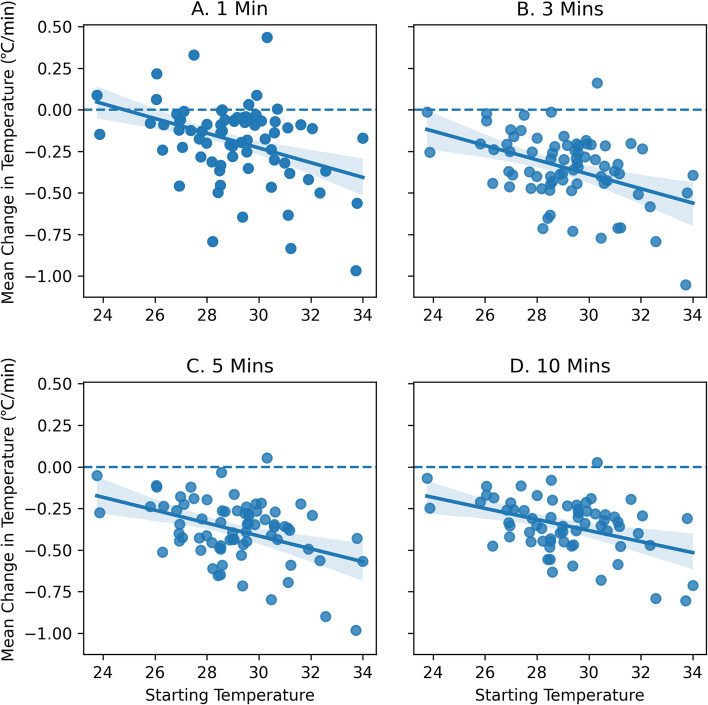
Relationship between starting temperature (°C) and maximum negative rate-of-change (°C/minute) at 1, 3, 5, and 10 minutes (Fig. 2A-D, respectively) using data from the training dataset. Dashed line indicates zero slope. Shaded band represents the 95% confidence interval

Phase one of algorithm development focused on characterizing the temperature dynamics that were associated with periods of sensor removal and subsequent donning. The decision to examine temperature rate-of-change as a parameter was driven by observation that a) there was often a delay between the absence of acceleration marking the potential start of a non-wear period and the absolute temperature threshold of 26 °C utilized by [25] and b) there were cases when the absolute threshold was not met despite known periods of sensor removal. There was also concern that differences in seasonal weather or climate would affect the accuracy of an absolute threshold for temperature [25]. To explore the association between rate of temperature change and known non-wear periods, regression analyses were conducted using a training dataset (see below) with starting temperature as the independent variable and negative rate-of-change (°C/minute) at 1, 3, 5, and 10 minutes as the targets, using all non-wear periods within the training dataset (Fig. 2). At one minute, the mean negative temperature rate-of-change was indistinguishable from normal temperature variations (i.e., values near zero). At three, five and ten minutes, however, temperature rates of change were appreciable. Based on these analyses, as detailed in the Results, temperature rate-of-change was deemed a viable feature for non-wear detection. Further, the five-minute window was selected given that confidence in the classification increased with longer window lengths but a significant proportion (12%) of the non-wear periods were less than 10 minutes in duration.

### Non-wear algorithm development

The DETACH algorithm was designed to improve the accuracy of non-wear start and end time detection compared to determining individual windows of data with predetermined lengths as either wear or non-wear within both the van Hees [41] and Zhou [25] algorithms. To establish the optimal parameters for detecting non-wear start and end periods, an open-source classification and regression trees (CART) decision tree classifier [42] with a depth of three was used to determine the best series of true-false conditions, based on given features, that would properly classify the data. Models were created for two different decision tree classifiers: one to detect the start of a non-wear period and another to detect the end. Both classifiers used the same features which included temperature rate-of-change as established in phase one of this study, as well as the previously established parameters of absolute temperature and one-minute rolling standard deviation for each of the three accelerometer axes used in one or both comparison algorithms [25, 41]. The depth hyperparameter of three was validated for both decision trees using cross-validation on the training set (see Supplementary File 1).

Fifteen of the 39 participants (38.5%) were used to train the classifiers with the remaining 24 participants used for testing. Data within the training dataset were first prepared by labelling all points as: wear, non-wear start (first 10 minutes), non-wear middle (beyond the first 10 minutes), or non-wear end (the 10 minutes following the end of a non-wear period). Wear and non-wear start data subsets were input into the “non-wear start” classifier while non-wear middle and non-wear end data subsets were input into the “non-wear end” classifier. The training dataset (*n* = 15 participants) contained 75 non-wear periods while the testing dataset (*n* = 24 participants) contained 111 non-wear periods. The results of the decision tree classifiers were used to create rules for detecting non-wear.

Lastly, to establish the final set of rules for the DETACH algorithm, results of the decision tree analysis were supplemented based on a) edge cases observed within the training data (e.g., low temperatures observed at start of some non-wear periods) and b) parameters reported in previously published non-wear papers, specifically using more than one axis of acceleration in the parameter set [26].

### Algorithm evaluation

Non-wear detection was compared for the DETACH algorithm and both the van Hees [11] and Zhou [25] algorithms using data from the 24 test participants. All algorithms were implemented using Python with one-second classification windows and then compared to the manually labelled non-wear periods from the reference dataset.

Accuracy (the fraction of correct predictions, both wear and non-wear, across all data), precision (the fraction of predicted non-wear time that was correctly identified), recall (the fraction of actual wear time correctly identified), and the F1 Score (the harmonic mean of the recall and precision) were computed for each of the three algorithms based on classification of algorithm predictions as true positive (TP), true negative (TN), false positive (FP), or false negative (FN) where TPs were correctly identified non-wear predictions and TNs were correctly identified wear predictions. Performance metrics were calculated for each participant using the formulae below:

$$\mathrm{Accuracy}=\left(\left(\mathrm{TP}+\mathrm{TN}\right)/\left(\mathrm{TP}+\mathrm{TN}+\mathrm{FP}+\mathrm{FN}\right)\right)$$

$$\mathrm{Precision}=\left(\mathrm{TP}/\left(\mathrm{TP}+\mathrm{FP}\right)\right)$$

$$\mathrm{Recall}=\left(\mathrm{TP}/\left(\mathrm{TP}+\mathrm{FN}\right)\right)$$

$$\mathrm{F}1\ \mathrm{score}=\left(2\times \left(\mathrm{precision}\times \mathrm{recall}\right)/\left(\mathrm{precision}+\mathrm{recall}\right)\right)$$

Outcomes were presented as an average with 95% confidence intervals. Analysis was conducted using Python (custom-coded or [42]).

## Results

**Fig. 3:**
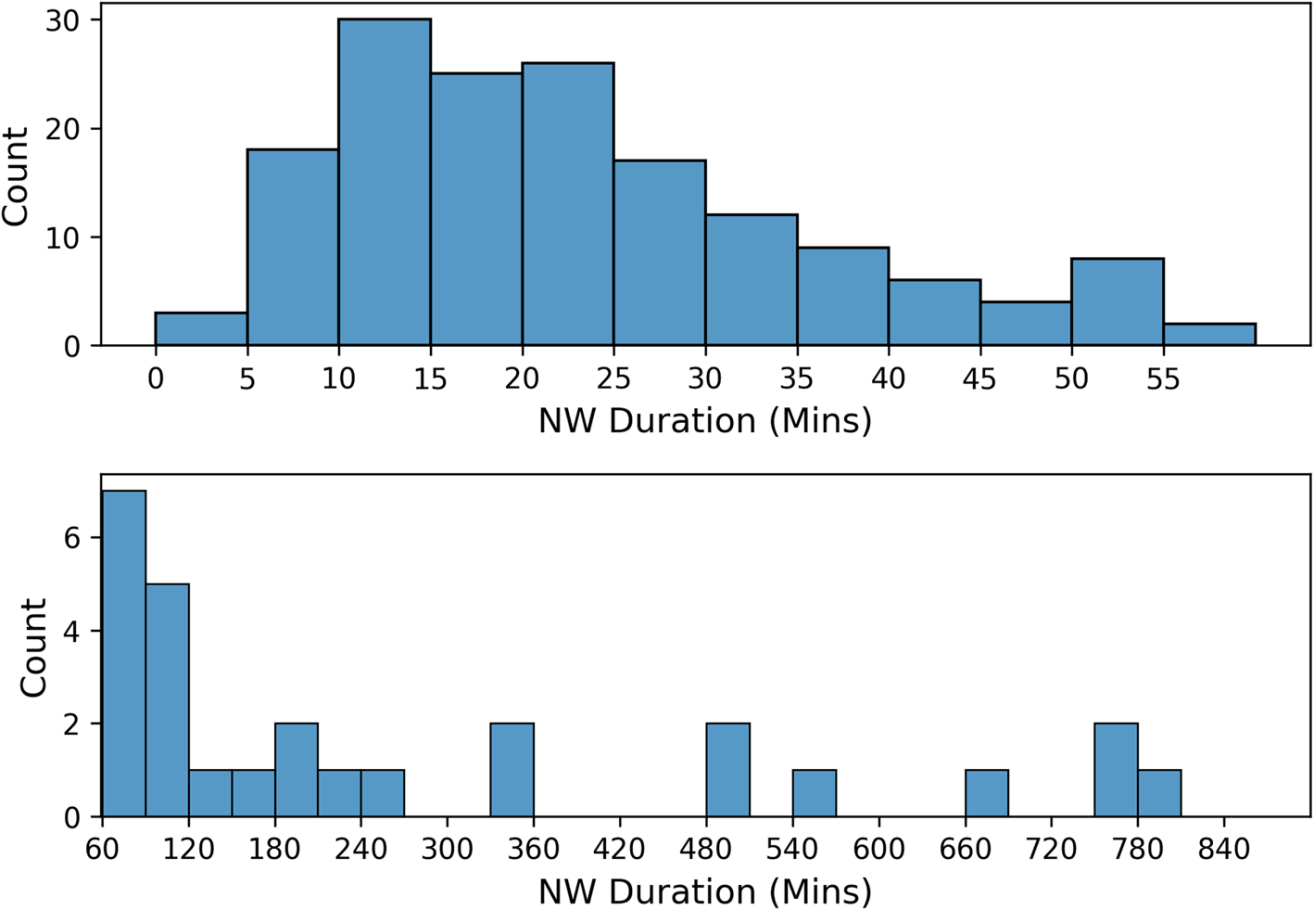
Distribution of non-wear periods by duration (minutes) with periods less than 60 minutes (top) and greater than 60 minutes (bottom)

A total of 186 non-wear periods were contained within the reference dataset with a median duration of 23 minutes (range: 3–808 minutes). Notably, 85.5% of these non-wear periods were less than 60 minutes. The distribution of the non-wear periods with respect to duration are presented in Fig. 3. On average, the temperature at the start of a non-wear period was 29.0 ± 2.0 °C with a range of 23.8–34.0 °C. For 70% of the non-wear periods, temperature remained above 26 °C five minutes into a non-wear period. For 20.4% of non-wear periods, temperature did not drop below the absolute threshold of 26 °C used by [25]. These instances occurred in 12 participants. The mean duration of these non-wear periods was 14.0 ± 9.6 minutes with a range of 1–45 minutes and a frequency of occurrence ranging from 10 to 100% of non-wear periods across participants.

### Rate-of-change in temperature as a non-wear parameter

**Fig. 4:**
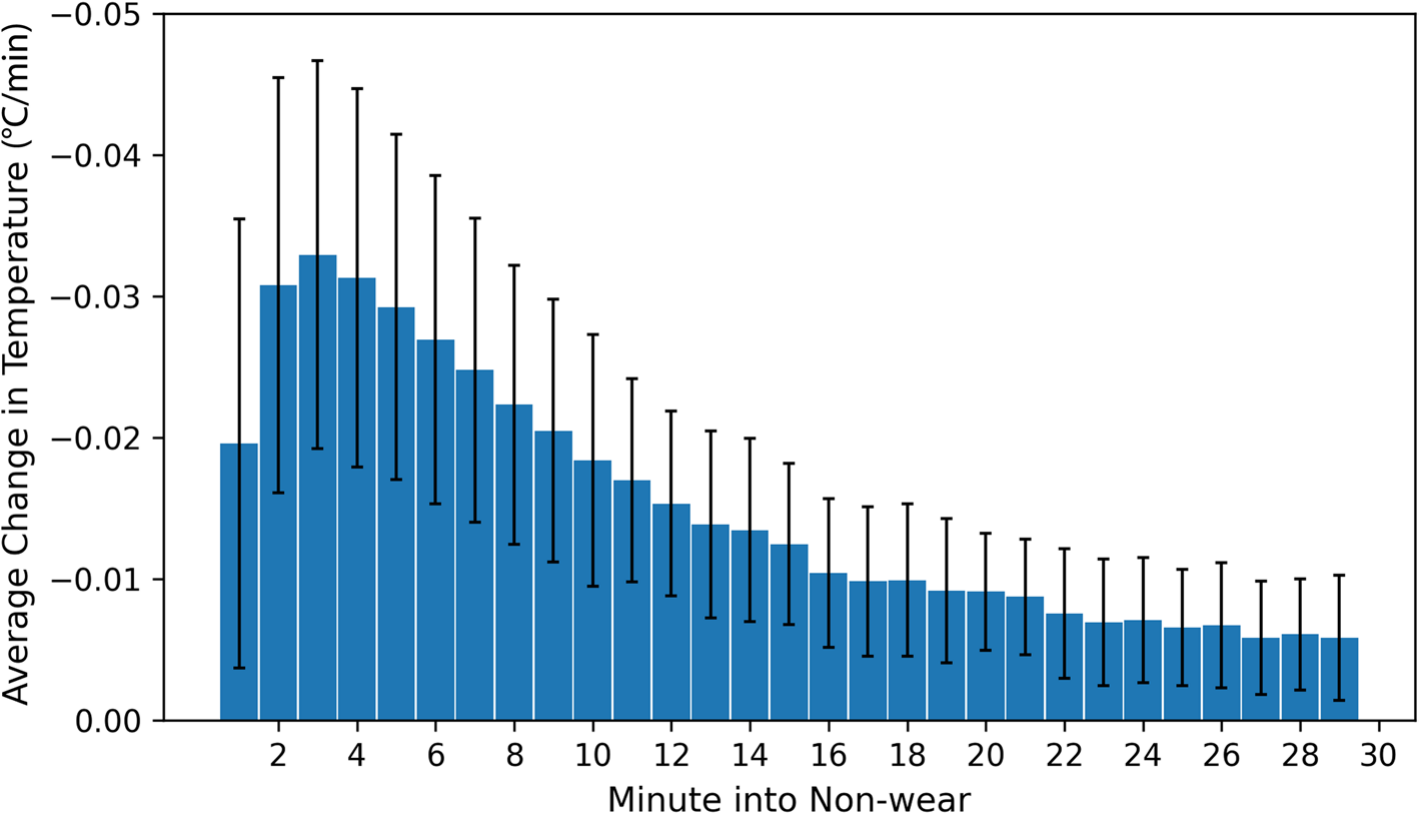
Average rate-of-change in temperature (°C/minute) by minute into non-wear period for data from the training dataset

Overall, 98.4% of non-wear periods included a decrease in temperature at the start of the non-wear period with an average rate-of-change in the following five minutes of − 0.40 ± 0.17 °C/minute. Similarly, 93.5% of non-wear periods included an increase in temperature at the end of the non-wear period with an average rate-of-change of 0.36 ± 0.21 °C/minute in the subsequent five minutes. Rates of change were comparable across non-wear periods of different lengths (Fig. 4).

In addition to understanding the dynamics of the temperature response to device removal and donning, we aimed to determine a threshold for rate-of-change in temperature that was robust enough to be associated with non-wear regardless of the starting temperature (non-wear start) or the ambient temperature (non-wear end). While the proposed shift to relative change in temperature from an absolute threshold was intended to limit false outcomes that could be related to ambient temperature, there was need to consider whether the rate-of-change was also impacted by ambient temperature (e.g., possibility that larger rates of change occurred when ambient temperature was lower). Regression analysis revealed that starting temperature impacted the rate-of-change in temperature with device removal, with higher starting temperatures leading to greater rates of change (*p* < .00001, R2 = 0.10). Starting temperature also impacted rate-of-change at the end of a non-wear period, with maximum rates of change greater when non-wear temperatures were low.

### Algorithm development: decision tree classification

The decision tree classifier determined that rate-of-change in temperature was an important parameter for identifying non-wear start time; specifically, a minimum decrease in temperature of 0.27 °C/minute. This condition was followed by the one-minute rolling SD of acceleration in the x-axis at ≤7 mg (relative inactivity), and then an absolute temperature of ≤29.5 °C. For non-wear end, the one-minute rolling SD of acceleration in two axes was determined to be an important parameter: specifically, that SD x-axis be ≥17 mg and SD z-axis be ≥138 mg for the data subscribed to the classifiers. The decision paths used to determine the start and end of a non-wear period are illustrated in Supplementary File 2. The non-wear start detection decision tree yielded an accuracy of 99.7% (range: 99.6 to 99.9%) on the test dataset where 99.4% of datapoints were classified as wear. The non-wear end detection decision tree yielded an accuracy of 98.8% (range: 96.7 to 100%) where 85.7% of samples were classified as non-wear middle.

### Final algorithm rules

Based on constraints of the CART approach and observations made via edge cases, the outputs of the decisions tree classifiers were supplemented to produce a more intuitive and robust algorithm that was not specific to GENEActiv devices [37]. Specifically, conditions of the decision tree analysis required that each axis of accelerometry be considered separately within both non-wear start and non-wear end. A recent paper by [26] however, demonstrated that using two or three axes within the parameter set optimizes non-wear detection. As such, the DETACH algorithm was refined to include acceleration features for multiple, non-specific, axes for both start and end of non-wear. Given the number of axes considered within the final set of rules, the threshold for SD of acceleration was increased slightly from 7 to 8 mg. Specifically, for non-wear start, SD of acceleration over the following minute for 2 or more axes was set to be < 8 mg. This was combined with the need for the SD of acceleration of 2 or more axes to be 8 mg for at least 90% of the following 5 minutes. The 90% requirement was intended to avoid cases where spurious movement occurring during non-wear (e.g., a device being bumped) would be misinterpreted as wear. Additionally, the rate-of-change in temperature criterion for non-wear start was modified slightly from − 0.27 to − 0.2 but the absolute temperature threshold remained < 30 °C (rounded). To detect a non-wear end event, SD of acceleration during the previous minute was set to be > 8 mg for all axes, combined with a SD of acceleration > 8 mg for two or more axes for at least 50% of the following 5-minute window. This SD condition for non-wear end is followed by the need for the 5-minute temperature rate-of-change to be > 0.1 °C/minute.

Visual inspection of non-wear periods in the test dataset that were not detected using the above pathways revealed cases in which the ambient temperature was close to the near-body temperature at the start of a non-wear period or rate-of-change didn’t meet the threshold of a minimum 0.2 °C decrease per minute. To address these possibilities, additional non-wear criteria focusing on absolute temperature were created to accompany the rate-of-change criteria. While maintaining the rules for accelerometry, the additional criteria serve to identify periods where temperature is below 26 °C for the start of a non-wear period or above 26 °C for non-wear end. Table 1 summarizes the non-wear rules based on decision tree classifiers, published literature, and edge case observations (algorithm available, see data availability statement).

**Table 1:** Evaluation measures for DETACH, Zhou [25], and van Hees [11] algorithms applied to the reference testing dataset. Data represent means (± 95% CI)

	Precision	Recall	F1-Score	Accuracy
**DETACH**	0.942 (0.844 to 1.000)	0.942 (0.883 to 1.000)	0.942 (0.880 to 1.000)	0.996 (0.994 to 1.000)
**Zhou**	0.538 (0.395 to 0.681)	0.824 (0.742 to 0.906)	0.651 (0.551 to 0.751)	0.971 (0.958 to 0.983)
**van Hees**	0.710 (0.554 to 0.866)	0.590 (0.451 to 0.728)	0.644 (0.535 to 0.753)	0.978 (0.973 to 0.984)

### Model evaluation

For all algorithms, data were examined in one-second classification windows for a total of 12,441,600 data points, of which 454,772 data points were marked as non-wear (contained within the 111 non-wear periods of the test dataset). The evaluation measures reported in Table 1 show high accuracy for all three methods however, the DETACH algorithm achieved better precision (positive predictive value) than the Zhou algorithm [25] and better recall (sensitivity) than the van Hees algorithm [11].

The high accuracy for all algorithms is largely due to success in detecting wear time which represented most of the data (non-wear averaged 2.25 ± 5.42% of data collection period across participants). The lower recall for the Van Hees algorithm [11] can be attributed to the longer interval length required for non-wear classification while the lower precision for the Zhou algorithm [25] can be accounted for by differences in criteria for this algorithm compared to the DETACH algorithm. Along with accelerometer criteria similar to [11], the Zhou algorithm [25] looked for absolute temperature values below 26 degrees. This absolute temperature criterion led the Zhou algorithm to falsely identify many short duration non-wear periods when there was low ambient temperature (e.g., if the device was worn outside in cold weather). Conversely, the van Hees accelerometer-only algorithm [11] with minimum duration non-wear of 60 minutes resulted in long periods of sedentary behavior being misidentified.

For the DETACH algorithm, wear time was correctly identified 99.8% of the time and misclassified 0.2% of the time. Further, the DETACH model correctly classified 94.2% of non-wear time and misclassified 5.8% of non-wear time as wear time. The low-temperature path was used for 6.1% of identified non-wear starts while the high-temperature path was used to uniquely identify 7.0% of non-wear ends (i.e., ends that were not identified by the rate-of-change in temperature pathway). Confusion matrix values are shown in Table 2.

**Table 2:** Confusion matrix for DETACH algorithm (values in minutes)

True Class (reference dataset)	Predicted Class	
	Negative (Wear)	Positive (Non-wear)
Negative	198,157	394
Positive	395	6436

The precision score was largely influenced by the low temperature path falsely detecting a non-wear start. Although accounting for a small proportion of total non-wear start detection (6%), these instances contributed to a large proportion of false positive outcomes (30%).

## Discussion

This paper presents a novel algorithm for identifying the start and end of non-wear periods by detecting temperature and acceleration changes characteristic of device removal and donning. The DETACH algorithm builds upon two commonly used non-wear algorithms: the accelerometry-only algorithm developed by [11] and the combined temperature and acceleration algorithm developed by [25], by adding a *rate-of-change* criterion for temperature. The DETACH algorithm performed well at a resolution of 5 minutes, with precision and recall that translated to improved F1-scores compared to these previous algorithms. As we move toward integrating remote, wearables-based monitoring methods into healthcare, there is critical need to ensure accurate non-wear detection. As noted, the consequence of misclassifying non-wear is an overestimation of sleep or sedentary behavior which can impact understanding of the volume and patterns of daily activities. Further, inaccurate information regarding compliance to sensor wear could have implications for the perceived utility of wearables for clinical application. It is worth reinforcing that the approach used in this study is specifically applicable to devices that include a tri-axial accelerometer and temperature sensor. These features are common to several frequently used clinical research devices (e.g., [37], [43]).

Correct detection of short non-wear periods is particularly important under conditions of extended device wear, as occurs during remote health monitoring, where occasional removals occur and can vary considerably in length. This study showed that 86% of the non-wear periods for a wrist-worn accelerometer were less than 60 minutes in duration, emphasizing the need to improve the resolution for non-wear detection afforded by commonly used non-wear algorithms (e.g., [11]), as well as the length of non-wear periods used for algorithm validation (e.g., [25]). Regarding the latter, although the Zhou algorithm [25] had no minimum length rule embedded in their non-wear algorithm, development of the algorithm was based on wear and non-wear reference periods of a minimum 15-minutes. The authors did note the possibility that the algorithm would not work well on shorter periods but suggested that such small errors would have a relatively small impact on population study outcomes [25]. Misclassification of short non-wear periods, however, may be significant at the individual level when considered in the context of clinical application, specifically if accumulated over time. This study successfully detected short non-wear periods with an algorithm that does not rely on minimum window lengths and uses improved logic for accurate detection of non-wear start and end times.

While the DETACH algorithm had excellent accuracy and precision, there were some situations in which temperature features did, or could, impact performance. Specifically, although the total number of false positives was minimal (6%), a proportion of these (30%) occurred when the low, absolute temperature pathway was used for non-wear start. Since only 6.1% of instances used this pathway, the false positive rate proved greater for it versus the temperature rate-of-change pathway. The decision to include the absolute temperature criterion for non-wear starts was based on a desire to address edge case instances where rate-of-change in temperature did not capture non-wear start. These tended to occur when the starting temperature was near 26 °C. Here, an assumption was made that wear temperature below 26 °C would be relatively uncommon, so that any sedentary time that occurred while the temperature was below this threshold would be considered non-wear. Notably, some participants exhibited a lower than typical wear temperature leading to false non-wear predictions within their data via the low-temperature pathway. Although this is a limitation of the current algorithm, it is important to note that overall, 93% of non-wear starts were detected using the rate-of-change in temperature pathway, avoiding this error.

Using rate-of-change in temperature takes advantage of the edge of each non-wear period; better defining the beginning of a non-wear period compared to Zhou’s algorithm [25], for example, which considers each datapoint as wear or non-wear based on an absolute temperature threshold of 26 °C. In the current study, the average non-wear start temperature was 29.0 °C (range of 23.8–34.0 degrees) and for 70% of non-wear periods, temperature remained above 26 °C five minutes into a non-wear period. In these cases, non-wear start classification using the absolute temperature threshold would lead to a delayed non-wear start classification or a missed non-wear period if the duration was short (i.e., 5 minutes). It is worth noting here that the accuracy and precision of non-wear detection using rate-of-change in temperature is impacted by instances when there is a rapid change in temperature accompanied by sedentary behavior such as when participants remove their wrist from under bedsheets during sleep. Since this scenario is most likely to occur overnight, consideration could be given to including specific time-of-day criteria (e.g., minimum 60-minute duration overnight) or using the non-wear detection algorithm in conjunction with a sleep detection algorithm.

The data used to develop and test the DETACH algorithm were captured from older adults living with NDD in a Canadian climate, over the course of several seasons, providing a broad range of ambient temperatures. The characteristics of the participants were distinct from the young children [22, 27] and healthy, young adults [6] who have typically been included in non-wear algorithm development studies. By contrast, the participants in this study engaged in relatively longer sedentary periods that have the potential to be misclassified as non-wear and who are likely to experience health benefits with even small increases in physical activity volume [32]. From a clinical perspective, these small changes may also be important for understanding disease trajectory and response to intervention.

Future work aims to investigate whether different wear locations and ambient temperatures impact the algorithm’s performance. Across studies, accelerometers are worn on body locations other than the wrist. As examples, in [25], children were instructed to wear watch-style accelerometers on their ankles, and in our own work, participants wore accelerometers in custom-made sleeves constructed from tensor wrap (i.e., no direct skin contact), as well as a chest worn device (see [16]). The current study showed some impact of starting temperature on rate-of-change in temperature, with a higher starting temperature linked to a more rapid decrease. This difference in starting temperature is attributable, in part, to the ambient temperature. Incorporating additional parameters, such as average daily temperature or current absolute temperature, into the rate-of-change threshold values has the potential to make the algorithm more robust. It is also worth exploring time varying changes in temperature within a person over extended periods of time so that person-specific adjustments can be made either to rate-of-change or absolute temperature threshold values. Accounting for the potential impact of these factors on performance of the DETACH algorithm will be important for optimizing its use across a range of scenarios.

## Conclusion

In summary, the DETACH algorithm presented in this study has several advantages over existing non-wear algorithms. Most importantly, the algorithm uses rate-of-change in temperature which makes it more broadly applicable to different settings and climates. Second, the algorithm can detect shorter non-wear periods which may be relevant in free-living scenarios when the data includes short removals (e.g., during bathing) as was observed in the study sample. Finally, the algorithm provides a simple and efficient model that does not require machine learning expertise. These features make the DETACH algorithm particularly relevant for clinical application.

## Supplementary Information

**Additional file 1: Supplementary File 1.** Results of hyperparameter cross-validation on the training dataset for non-wear start (top) and non-wear end (bottom).

**Additional file 2: Supplementary File 2.** Results of decision tree analysis for non-wear start (top) and non-wear end (bottom).

## Data Availability

Data are available to researchers for purposes of reproducing the results or replicating the procedures. Available data files will be released through Brain-CODE (www.braincode.ca), accessible through a data access request. Please see the Ontario Brain Institute website for information on when the data will be released and how to access the data (https://braininstitute.ca/). Source code for the version of the DETACH algorithm referenced in this manuscript is preserved at 10.5281/zenodo.6557609 and the most recent version can be found at https://github.com/nimbal/vertdetach. For details, contact: kit.beyer@uwaterloo.ca.

## Abbreviations

CART: Classification and regression trees
CTA: Combined temperature and acceleration
DETACH: Device temperature and acceleration change
EDF: European Data Format
FN: False negative
FP: False positive
NDD: Neurodegenerative disease
ONDRI: Ontario Neurodegenerative Disease Research Initiative
ReMiNDD: Remote Monitoring in Neurodegenerative Disease
TN: True negative
TP: True positive
