# Supplementary material for: Detecting accelerometer non-wear periods using change in acceleration combined with rate-of-change in temperature
Source: BMC Med Res Methodol. 2022 May 20;22:147. doi: 10.1186/s12874-022-01633-6 (PMC9123693; doi:10.1186/s12874-022-01633-6)

Supplementary File 1: Results of hyperparameter cross-validation on the training dataset for non-wear start (top) and non-wear end (bottom)


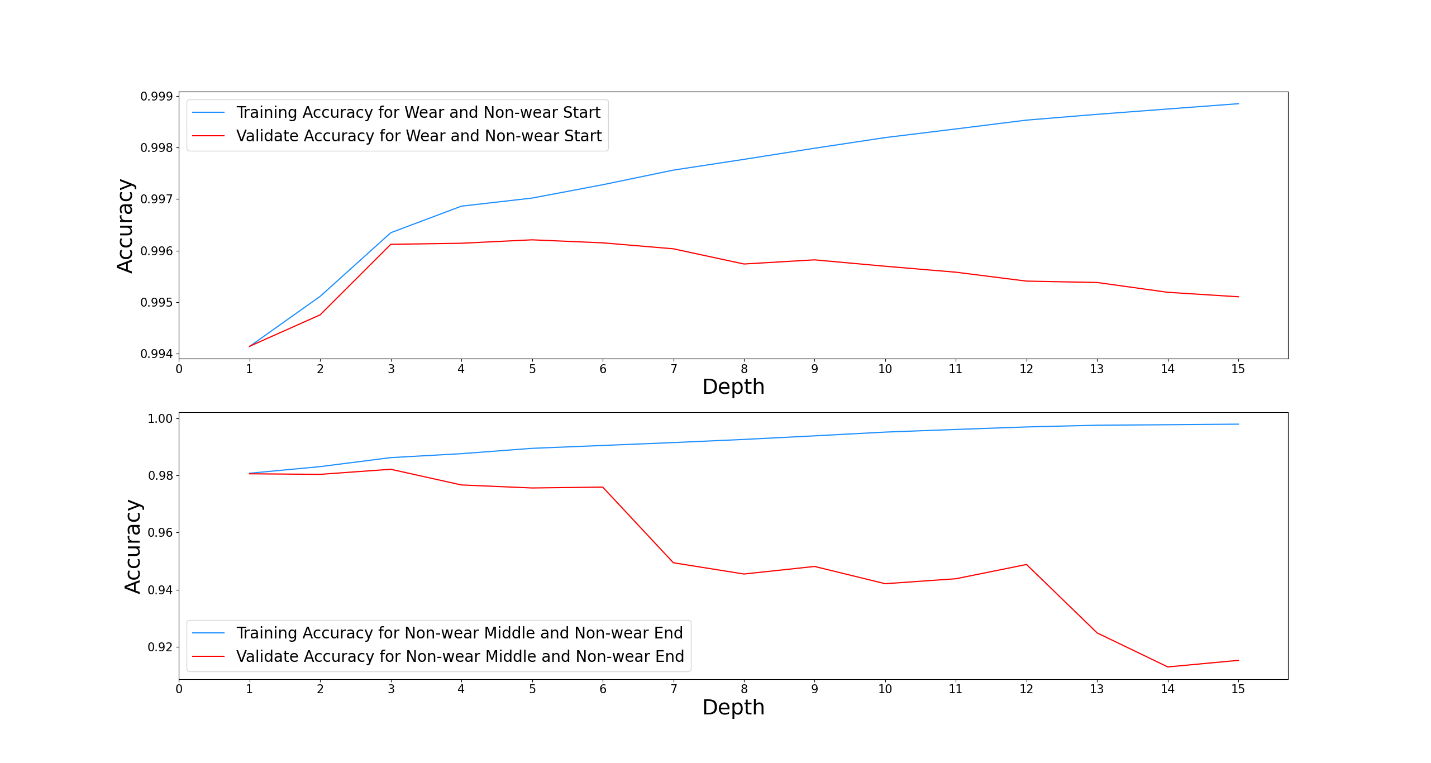

Supplement: Supplementary file 1 — Additional file 1: Supplementary File 1. Results of hyperparameter cross-validation on the training dataset for non-wear start (top) and non-wear end (bottom). [file 12874_2022_1633_MOESM1_ESM.docx]
