# Supplementary material for: Detecting accelerometer non-wear periods using change in acceleration combined with rate-of-change in temperature
Source: BMC Med Res Methodol. 2022 May 20;22:147. doi: 10.1186/s12874-022-01633-6 (PMC9123693; doi:10.1186/s12874-022-01633-6)

Supplementary File 2: Results of decision tree analysis for non-wear start (top) and non-wear end (bottom)


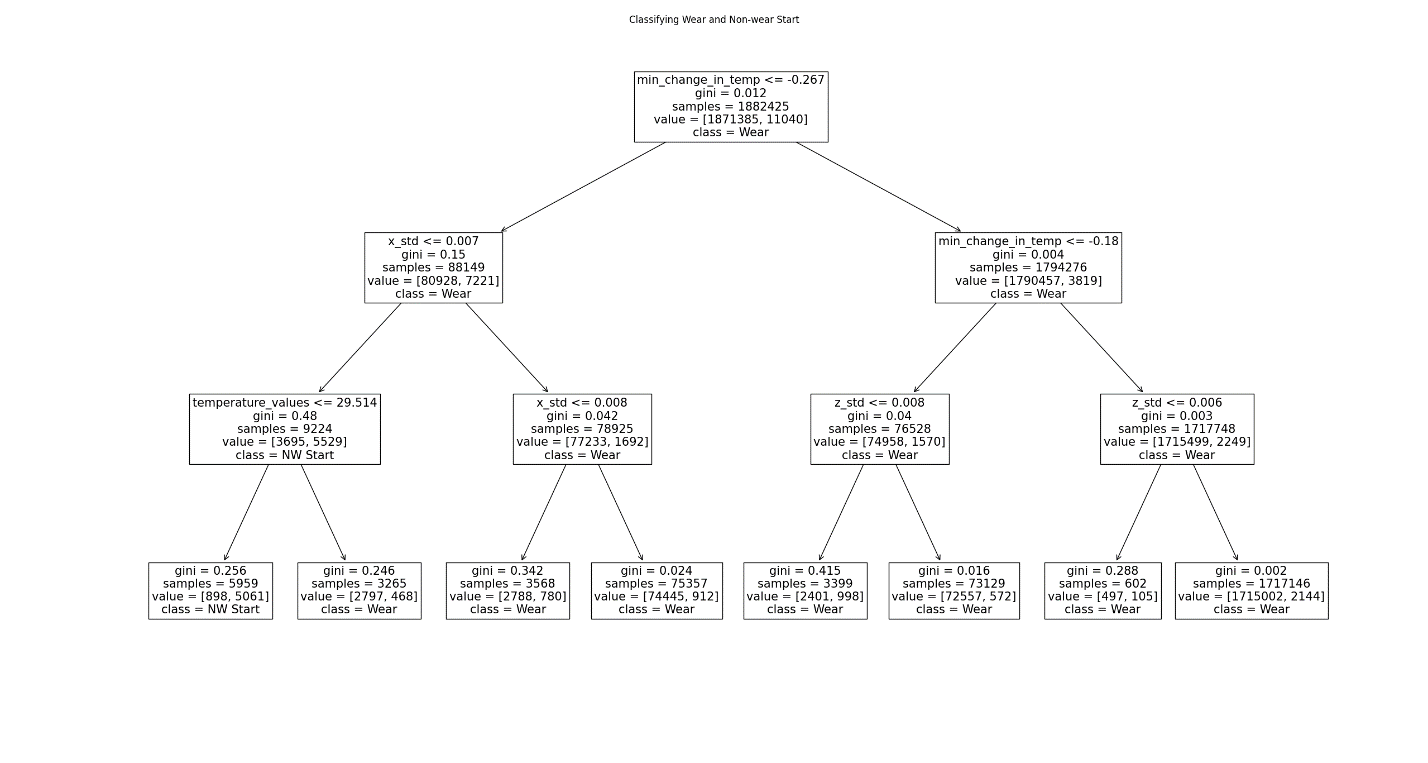


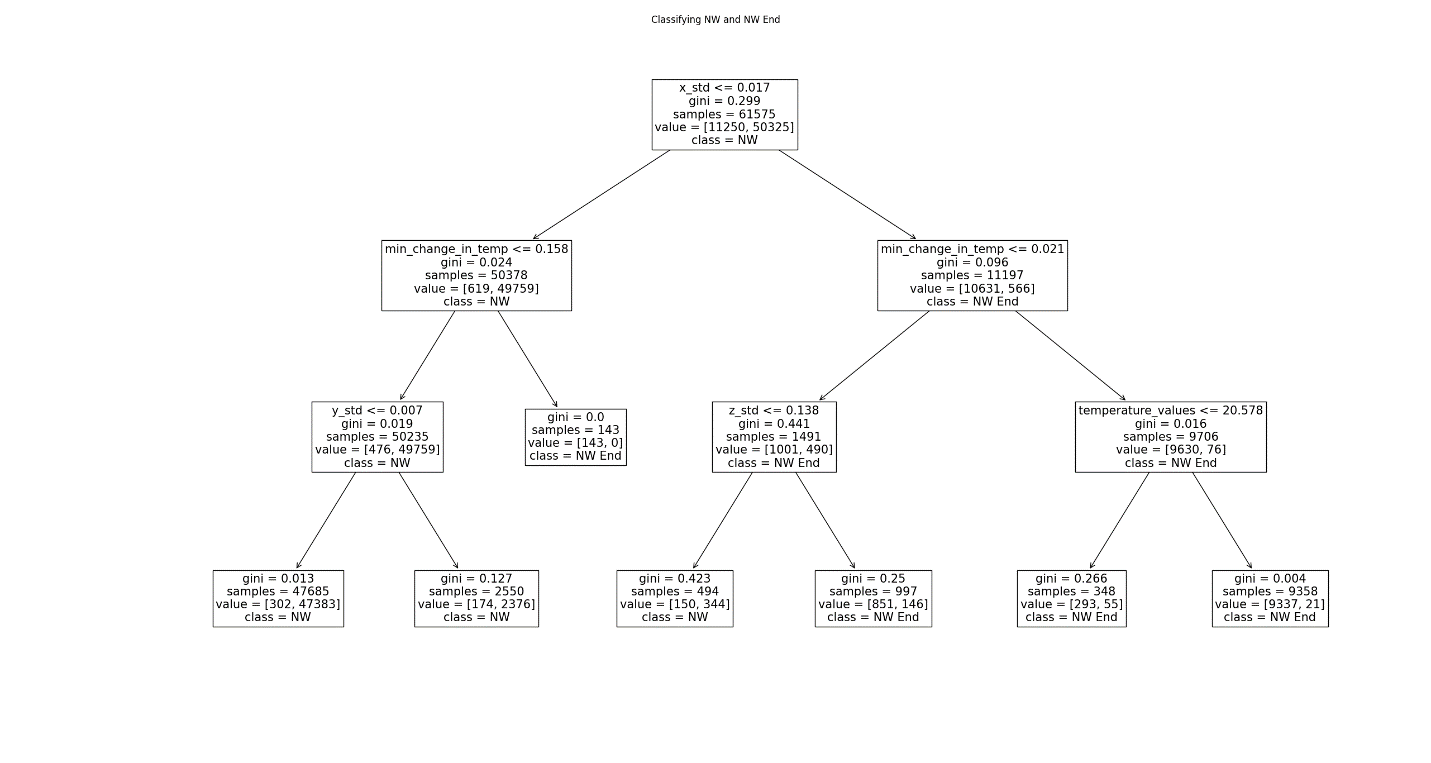

Supplement: Supplementary file 2 — Additional file 2: Supplementary File 2. Results of decision tree analysis for non-wear start (top) and non-wear end (bottom). [file 12874_2022_1633_MOESM2_ESM.docx]
